# Clinicopathological study of 1000 biopsied gingival lesions among dental outpatients: a 22-year retrospective study

**DOI:** 10.1186/s12903-022-02192-4

**Published:** 2022-04-29

**Authors:** Mohammad-Salar Montazer Lotf-Elahi, Golnoush Farzinnia, Zohreh Jaafari-Ashkavandi

**Affiliations:** 1 Department of Oral and Maxillofacial Pathology, School of Dentistry, Shiraz University of Medical Sciences, Shiraz, Iran; 2 Oral and Dental Disease Research Center, School of Dentistry, Shiraz University of Medical Sciences, Shiraz, Iran

**Keywords:** Gingiva, Biopsy, Histopathology, Reactive lesion, Squamous cell carcinoma, Pyogenic granuloma

## Abstract

**Background:**

Up to now, limited research has been done on a significant number of cases with all types of gingival lesion. Besides, the available literature does not provide reliable data on the epidemiology of gingival lesions, especially non-reactive lesions. Thus, the present study aimed to analyze the frequency and distribution of gingival lesions in an Iranian population.

**Methods:**

This retrospective study was conducted on 1000 gingival biopsy samples during 22 years. All lesion types were evaluated in terms of location, clinical signs and symptoms, and patients’ age and gender. The data were analyzed using descriptive statistics and chi-square test.

**Results:**

Out of the 5284 oral lesions, 1000 (18.92%) gingival lesions were detected, with a female dominance (64.06%). The incidence peak (35.6%) was observed in the third and fourth decades. Non-neoplastic lesions accounted for 92.4% of the cases. The most common reports were related to reactive lesions (71.8%), with the highest prevalence being related to pyogenic granuloma. Additionally, oral squamous cell carcinoma (OSCC) was the most common neoplasm, and exophytic changes and color changes were the most frequent clinical signs.

**Conclusions:**

The study findings indicated the high prevalence of gingival pathological lesions. Although most biopsies were reactive in nature, a few cases were malignant, which must be considered by practitioners. Further research is needed to achieve a clear impression about non-neoplastic lesions so as to develop more helpful oral health planning.

## Background

Gingiva has long been focused by many clinicians as one of the most prevalent sites for numerous diseases affecting oral health [1]. Although the majority of gingival lesions are inflammatory diseases caused by dental plaque biofilms, gingiva can be involved by a number of neoplastic or non-neoplastic diseases with etiologies other than bacterial biofilms [2, 3]. Non-neoplastic lesions are usually reactive in response to chronic irritations; i.e., Pyogenic Granuloma (PG), fibroma, and Peripheral Ossifying Fibroma (POF) [4]. Some systemic diseases and immunologic reactions can also involve the gingiva. In addition, true neoplasms, both benign and malignant, are found frequently in this location [3, 5]. These tumors originate from epithelial or mesenchymal tissues of the gingiva as well as from the adjacent soft or jaw tissues [1]. For better management of gingival lesions, proper diagnosis is critical due to their different implications on etiology, clinical behavior, and treatment requirements. Although clinical features are used to diagnose gingival diseases, histologic examination may be required to establish the diagnosis [6, 7].

Up to now, limited epidemiological studies have been done on all gingival lesions among various populations [8–11]. Most of these studies have evaluated few cases, focusing on reactive lesions. Several studies reported the high prevalence of non-neoplastic lesions in the gingiva in comparison with neoplasms and indicated that PG was the most frequent lesion in the gingiva. In addition, benign neoplastic lesions such as fibroma and fibrolipoma accounted for a high percentage of tumors [10, 12]. Furthermore, oral squamous cell carcinoma (OSCC) was found as a common non-plaque-induced lesion in this location [3]. Hanisch et al. [13] revealed that in 14% of rare diseases with an orofacial manifestation, gingiva was involved not only with a gingivitis and periodontitis presentation, but also as a gingival hyperplasia related to an underlying disease. Another study indicated that gingiva was the most common site of involvement by peripheral soft connective tissue lesions in an Iranian population [14]. Moreover, one study showed that geographical variations might influence the prevalence of different gingival lesions [11].

To the best of our knowledge, no long-term study has been done on the distribution and frequency of gingival lesions (neoplastic, inflammatory, plaque induced, and non-plaque induced) in Iran. Hence, the present study aims to determine the prevalence and distribution of all types of gingival lesion in an Iranian population over a long period. Demographic data and incidence of lesions can help practitioners provide more accurate differential diagnosis and manage patients more efficiently.

## Methods

This retrospective study was performed at the Oral and Maxillofacial Pathology Department, Faculty of Dentistry, Shiraz University of Medical Sciences. The study was concordant with all relevant principles of the Helsinki Declaration and was approved by the Ethics Committee of Shiraz University of Medical Sciences, Shiraz, Iran (IR.SUMS.DENTAL.REC.1398.106). Additionally, informed consent forms were obtained from all the participants. Out of a total of 5284 biopsy records over a 22-year period (1996–2017), all gingival samples were retrieved. Patients’ demographic data (age and gender), location of the lesion, clinical signs or symptoms (including exophytic changes, color changes, and ulcer), and the final histopathological diagnosis were recorded. The samples without a definite histological diagnosis due to inappropriate biopsied samples or lack of clinical data were excluded from the research. Regarding location, the gingival lesions were categorized as anterior (related to incisors and canines), posterior (related to premolar and molar teeth), maxillary, and mandibular. Based on the text book of Oral and Maxillofacial Pathology with slight modifications [15], considering the origin, nature, and etiologic factors of the lesions, the defects were divided into nine groups as follows: soft tissue lesions (reactive lesions, benign mesenchymal neoplasms, and malignant mesenchymal neoplasms), epithelial lesions (benign lesions and malignant neoplasms), immune-mediated diseases, salivary gland pathologies (non-neoplastic, benign neoplastic, and malignant neoplastic lesions), periodontal lesions, odontogenic cysts and tumors, hematopoietic lesions, physical and chemical irritation, and non-specific lesions (the gingival lesions without any definite diagnosis). The lesions with a frequency of one case were placed in the “others” group.

### Statistical analysis

All data obtained from the patients’ records were entered into the SPSS 23 software and were analyzed using descriptive statistics and chi-square test. P < 0.05 was considered statistically significant.

## Results

Out of the 5284 oral lesions, 1000 (18.92%) were found in the gingiva. Among the patients, 640 (64.06%) were female, 359 (35.93%) were male, and gender was not mentioned in one case. The mean age of the patients was 38.70 ± 18.28 years, ranging from 1 to 90 years. The lesions were most frequently observed in the 20–39 age group (36.7%). The youngest patient was a one-year-old boy with PG and the oldest one was a 90-year-old male with the same diagnosis. The frequency of different lesions based on the patients’ age and gender has been summarized in Table 1. The “others” group, included lipoma, schwannoma, oral focal mucinosis, fibrous histiocytoma, lupus erythematosus, mucoepidermoid carcinoma, adenocarcinoma, gingival fibromatosis, plasmocytoma, gingival hyperplasia, gingival cyst of the adult, langerhans cell histiocytosis, and hematoma formation.

**Table 1:** The distribution of various gingival lesions based on the patients’ age and gender

Lesion	Frequency N (%)	Age (Mean ± SD)	M:F
Irritation fibroma	85 (8.5)	35.62 ± 15.34	32:53
POF	167 (16.7)	31.30 ± 15.66	66:101
IFH	51 (5.1)	58.88 ± 13.06	15:36
Pyogenic granuloma	246 (24.6)	35.50 ± 17.01	81:165
PGCG	150 (15.0)	38.36 ± 20.64	75:74
Epulis granulomatosa	7 (0.7)	47.16 ± 29.43	3:4
Giant cell fibroma	12 (1.2)	34.09 ± 23.91	8:4
Neurofibroma	19 (1.9)	40.11 ± 18.99	7:12
Hemangioma	3 (0.3)	27.00 ± 22.51	2:1
Malignant mesenchymal tumor	7 (0.7)	43.42 ± 18.20	3:4
Oral melanotic macule	13 (1.3)	39.41 ± 9.36	3:10
Hyperkeratosis	22 (2.2)	46.95 ± 16.96	10:12
Epithelial dysplasia	5 (0.5)	53.80 ± 7.25	2:3
Squamous papilloma	4 (0.4)	37.75 ± 8.46	2:2
SCC	26 (2.6)	59.7 ± 14.08	14:12
Melanoma	2 (0.2)	55 ± 0.00	1:1
Undifferentiated carcinoma	2 (0.2)	55.50 ± 13.43	2:0
Lichenoid reaction	43 (4.3)	48.04 ± 11.76	1:42
Pemphigoid	6 (0.6)	49.83 ± 13.49	0:6
Lichen planus	34 (3.4)	43.03 ± 12.18	7:27
Pemphigus	6 (0.6)	37.66 ± 10.63	0:6
Plasma cell gingivitis	4 (0.4)	32.75 ± 21.46	0:4
Periodontitis/ Gingivitis	5 (0.5)	27.60 ± 6.42	0:5
Peripheral odon. fibroma	3 (0.3)	22.00 ± 8.71	0:3
Lymphoma	3 (0.3)	55.33 ± 30.17	1:2
Exogenous pigmentation	5 (0.5)	32.00 ± 14.66	1:4
Non-specific lesions	57 (5.7)	39.63 ± 17.24	22:35
Others	13 (1.3)	27.34 ± 18.49	0:13
Total	1000 (100)	38.72 ± 18.30	359:640

*POF* peripheral ossifying fibroma, *IFH* inflammatory fibrous hyperplasia, *PGCG* peripheral giant cell granuloma, *SCC* squamous cell carcinoma, *Odon* odontogenic

Reactive soft tissue lesions (71.8%) were the most frequent, with females accounting for 60.94% of the cases. Immune-mediated lesions (19.2%) were the second most common lesions of the gingiva, with a clear female tendency (91.11% of all the cases). Distribution of gingival lesions according to the patients’ gender and age has been presented in Table 2. Accordingly, reactive soft tissue lesions, immune-related lesions, and periodontal lesions were significantly more prevalent in females (p = 0.001, chi-square test), while malignant epithelial neoplasms were significantly more prevalent among males (p = 0.027). However, gender tendency was not significant in other groups (p > 0.05).

**Table 2:** Distribution of each category of gingival lesions according to the patients’ gender and age

	Male (n = 359)	Female (n = 640)	Total (n = 999)	P value	Mean ± SD
Reactive soft tissue lesions	280 (28)	437 (43.7)	717 (71.8)	0.001*	36.92 ± 18.53
Benign mesenchymal neoplasms	9 (0.9)	17 (1.7)	26 (2.6)	0.948	37.32 ± 19.09
Malignant mesenchymal tumors	3 (0.3)	4 (0.4)	7 (0.7)	0.990	43.42 ± 18.20
Benign epithelial lesions	17 (1.7)	27 (2.7)	44 (4.4)	0.825	44.68 ± 14.05
Malignant epithelial tumors	17 (1.7)	13 (1.3)	30 (3)	0.027*	59.13 ± 13.40
Immune-mediated diseases	8 (0.8)	82 (8.2)	90 (9)	0.001*	45.65 ± 12.18
Salivary gland malignancies	0	2 (0.2)	2 (0.2)	0.747	41.00 ± 15.55
Periodontal lesions	0	12 (1.2)	12 (1.2)	0.021*	27.08 ± 15.29
Odontogenic lesions	0	4 (0.4)	4 (0.4)	0.327	22.50 ± 7.18
Hematopoietic lesions	1 (0.1)	3 (0.3)	4 (0.4)	0.947	38.12 ± 34.68
Physical and chemical irritation	2 (0.2)	4 (0.4)	6 (0.6)	0.769	28.00 ± 16.37
Non-specific lesions	22 (2.2)	35 (3.5)	57 (5.7)	0.77	39.63 ± 17.24

^*^Significant difference

**Fig. 1:**
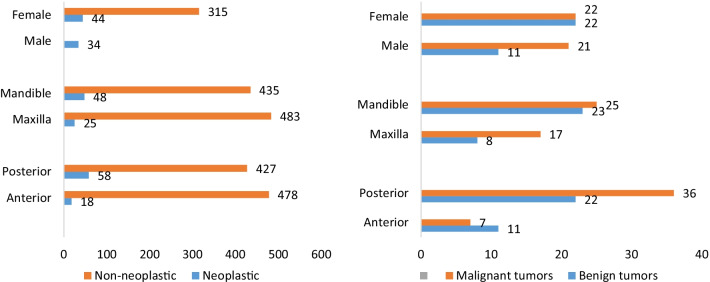
Patients’ gender and location: neoplastic and non-neoplastic lesions (left), benign and malignant tumors (right)

**Fig. 2:**
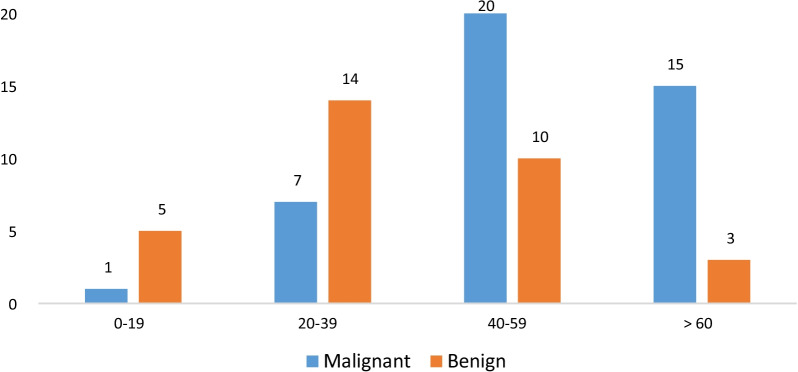
Distribution of benign and malignant tumors in different age groups

**Fig. 3:**
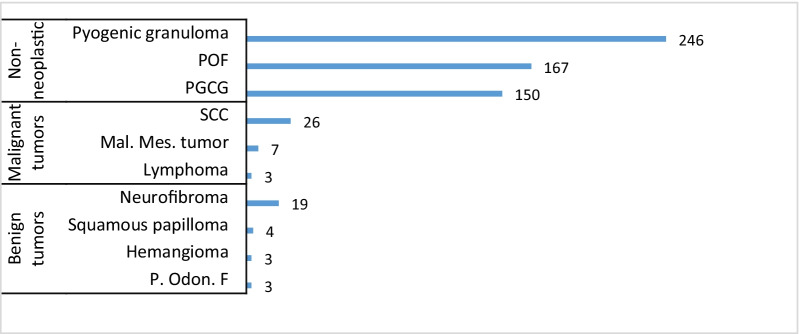
Frequency of three most common lesions in neoplastic and non-neoplastic groups. POF, peripheral ossifying fibroma, PGCG, peripheral giant cell granuloma, SCC, squamous cell carcinoma, Mal. Mes., malignant mesenchymal, Odon, odontogenic

Non-neoplastic lesions (92.4%), benign neoplasms (3.30%), and malignant neoplasms (4.30%) accounted for most frequent biopsied gingival lesions. The most common neoplasms were OSCC (26 cases), neurofibroma (19 cases), and malignant mesenchymal tumors (7 cases). Malignant tumors (including lymphoma, epithelial and mesenchymal tumors) showed an equal incidence in both genders, while most benign tumors were found in females (66%). Further details on the patients’ age and gender, the most common lesions, and locations of neoplastic and non-neoplastic lesions have been shown in Figs. 1, 2, 3.

Based on the results, the lesions were reported in the maxilla (50.5%) and the mandible (47.9%) and 1.6% of the lesions involved both jaws. Moreover, anterior (48.1%) and posterior (47%) regions of the jaws were reported with an approximately equal incidence and 1.5% of the lesions involved both anterior and posterior parts. The anterior part of the maxilla (29%) and the posterior part of the mandible (27.3%) were the most common sites of the lesions. Most neoplastic lesions involved the mandibular gingiva, while non-neoplastic lesions were more frequently found in the maxilla (p = 0.003, chi-square test). Besides, more neoplastic lesions were reported in the posterior part of the jaws, while more non-neoplastic lesions were found in the anterior part with a significant difference (p = 0.002).

Exophytic changes (51.92%), exophytic changes accompanied by ulceration (30.86%), and color changes (12.72%), were the most frequent clinical signs. White and/or red color changes were mostly associated with immune-related lesions such as oral lichen planus, and hyperkeratosis. Moreover, a few cases with brown discoloration were found in association with melanotic lesions especially oral melanotic macules. Additionally, ulceration was the most frequent clinical sign recorded among the patients with malignancies (in 40% of the malignant cases).

## Discussion

Up to now, few studies have assessed the prevalence and distribution of all gingival lesions in different countries [2, 4, 7, 16]. According to the present study results, gingival lesions accounted for 18.92% of all the cases. This rate was reported as 9.5% among 1248 oral and maxillofacial biopsies in a Saudi Arabian population [6]. This discrepancy could be due to the differences in ethnic factors, geographical characteristics, study methodology, and sample size.

In the current study, the majority of the gingival biopsies (92.4%) had a non-neoplastic nature with a high rate of PG, which was consistent with the findings of other studies conducted on the issue [8, 10–12]. Zarei et al. [17] investigated a series of 172 cases and reported the clinical aspects of reactive hyperplasia of oral cavity amongst Iranian people. They confirmed that the clinical characteristics of reactive hyperplasia in Iranians were mostly consistent with those reported by other researchers. In a six-year retrospective study on 244 gingival biopsies by Shamim et al. [10] in southern India, non-neoplastic lesions made up 75.5% of the cases with PG. Neoplasms were also found in 24.5% of the specimens (6.5% malignant and 18% benign). OSCC and POF were the most frequent malignant and benign neoplasms, respectively. Similarly, Manjunatha et al. [12] conducted a retrospective study on 106 gingival biopsies over five years in an Indian population and found non-neoplastic lesions as the most frequent cases (73.58%), with PG being the most frequent lesion (38.46%). In addition, benign neoplastic lesions accounted for 92.85% of neoplasms, with a high prevalence of fibroma (30.76%) and fibrolipoma (26.92%). Some studies have used different classifications and considered some reactive lesions in the group of neoplastic lesions, thereby reporting a higher incidence of neoplasms [8, 18].

The present study findings demonstrated that the prevalence of malignant neoplastic lesions (4.3%) was slightly higher than that of benign tumors (3.3%), which was in line with the findings of other studies carried out in Italy, India, and Chile [2, 8, 19]. Most studies indicated the prevalence rate of about 2–8% for gingival malignant neoplasms [2, 6, 10, 12, 18, 19]. However, two studies reported this measure as 33.17% and 30% among non-dental plaque-induced gingival lesions in Chinese [3] and Indian [8] populations, respectively. This revealed the importance of precisely evaluating gingival lesions and finding the possible initiating factors. Furthermore, OSCC was the most common type of cancer in the present research as well as in the previous studies [2, 3, 6, 8, 10, 12, 17–20], accounting for 2.6% of all the lesions. This emphasized the importance of enhancement of oral surgeons’ knowledge as well as performance of careful clinical examinations for early detection and diagnosis of OSCC.

In the current study, females were more frequently affected by gingival lesions (64.06%) compared to male patients (35.93%), which was in agreement with the findings of other retrospective studies [2, 8, 9, 12, 18]. Reactive soft tissue lesions were also more common among females, which was similar to the findings of the previous studies [2, 4, 12, 21, 22]. This could be attributed to the effect of hormonal factors as well as to the thinner and more sensitive gingiva of females. However, malignant epithelial lesions were more frequent amongst males and OSCC was the main reported cancer, which was in line with the studies carried out in Chile, India, and Italy [2, 12, 19]. The present study findings showed OSCC with a male predominance, which was consistent with the results obtained in other studies [2, 6, 8, 12, 15]. OSCC is the most common oral cancer, which is mostly associated with smoking. Nonetheless, this association is less prominent in the gingiva [15]. In all the aforementioned studies, the number of OSCC cases was very small and oral cancer was most often reported in the mandibular gingiva [8, 12, 18].

In the current research, the peak incidence of all the lesions occurred in the third and fourth decades of life. In other words, these ages were associated with a high incidence of reactive lesions and benign neoplasms. Nevertheless, the incidence of neoplastic lesions, especially malignant ones, was higher in the fifth decade of life. The peak incidence of gingival lesions was also reported in the third and fourth decades by other researchers [8, 9, 18]. In the present investigation, gingival lesions were found in the upper and lower gingiva as well as in the posterior and anterior parts with a similar distribution, which was concordant with the results of the studies conducted in India and China [8, 19]. On the other hand, some researchers found the majority of biopsies in the maxilla [2, 12, 18]. It is worth mentioning that most of these studies evaluated a small number of lesions compared to the present study. Moreover, neoplastic lesions were observed in the posterior parts of the jaws as well as in the mandible, while non-neoplastic lesions were more common in the anterior areas of the jaws and the maxilla. Likewise, Kamath et al. [8] and Manjunatha et al. [12] disclosed that neoplastic and non-neoplastic lesions were more prevalent in the mandible and the maxilla, respectively. This finding should be considered for presenting the appropriate differential diagnosis of gingival pathologies.

In the present study, the most common clinical symptoms were exophytic changes with or without ulceration, both of which were significantly associated with reactive soft tissue lesions. Another frequent clinical symptom was color changes without swelling, which were mostly associated with immune-related lesions such as oral lichen planus, as white and red lesions. In malignant lesions, ulcer and exophytic changes were observed more frequently compared to the other signs and symptoms. This represented the need to pay attention to oral ulcers, especially in patients without a significant irritation factor. Similarly, the results of the research carried out by Gambino et al. [23] on 788 gingival biopsies demonstrated that the most frequently detected symptoms were exophytic lesions followed by color changes (white lesions, red lesions, and pigmented lesions), erosions, and ulcers. They also indicated that ulcerative lesions were positively related to OSCC.

This study had some limitations. It was difficult to compare studies from different nations due to the differences in the study design and classification of lesions as well as the limited number of cases, especially in non-plaque-related lesions. However, this research was the first report of the frequency and clinicopathology of biopsied gingival lesions in an Iranian population.

## Conclusions

The results of the present study showed a high prevalence of gingival lesions among oral biopsies with a female tendency. Reactive lesions, mostly PG, were the most frequent biopsied lesions. Moreover, malignant neoplasms, mainly OSCC, were more common compared to benign tumors. The nature of lesions in the anterior gingiva was different from those in the posterior region. Thus, further studies are recommended to be conducted in other dental centers to make a clear impression about the prevalence and features of non-reactive gingival lesions. These studies can provide valuable data for more accurate health planning and patient management.

## Data Availability

All data analyzed during this study are included in this published article.

## Abbreviations

POF: Peripheral ossifying fibroma
PG: Pyogenic granuloma
OSCC: Oral squamous cell carcinoma
